# Supplementary figures and images for: Investigation of Intestinal Microbes of Five Zokor Species Based on 16S rRNA Sequences
Source: Microorganisms. 2024 Dec 26;13(1):27. doi: 10.3390/microorganisms13010027 (PMC11767591; doi:10.3390/microorganisms13010027)

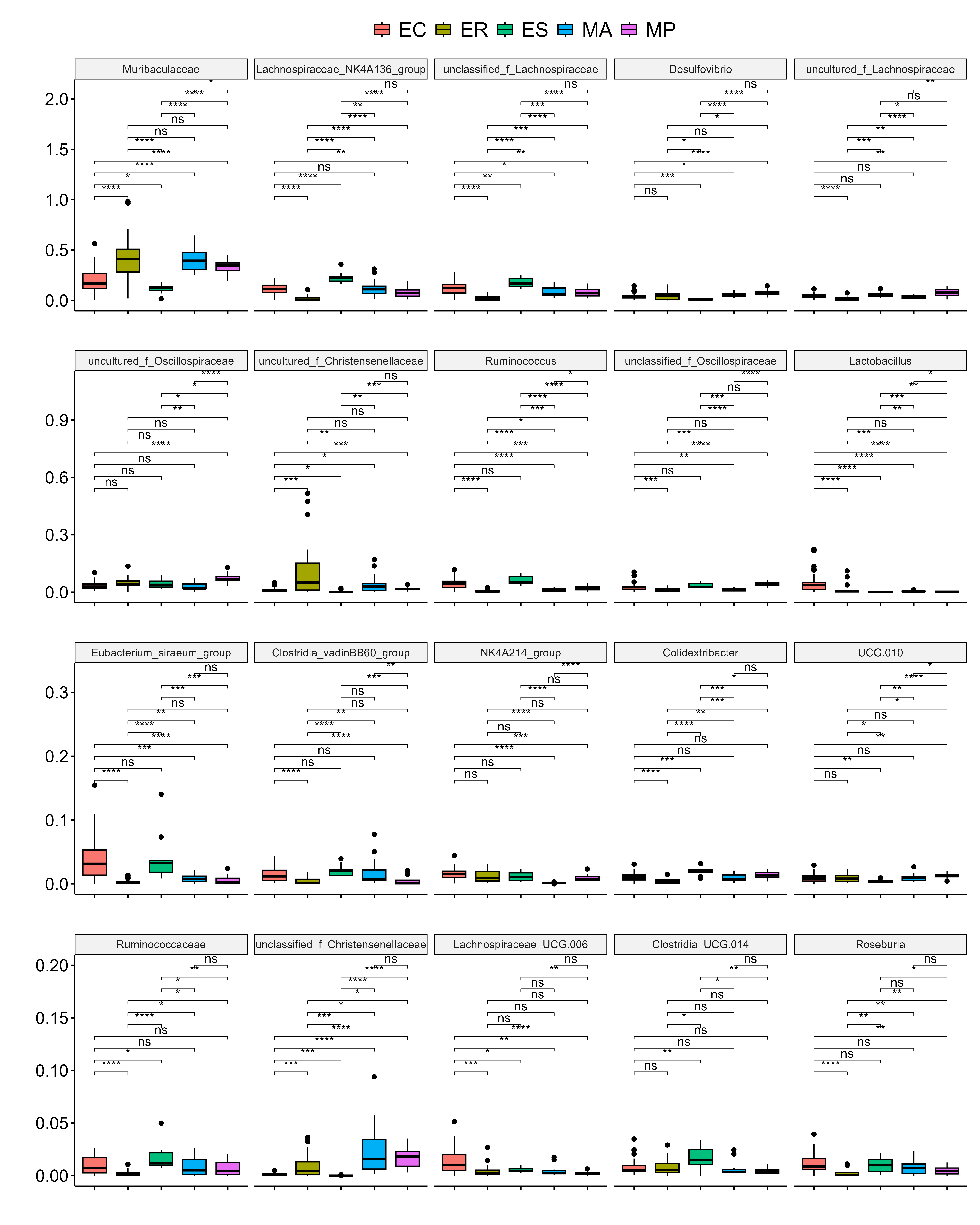

Supplement: Supplementary file 1 [file microorganisms-13-00027-s001.zip › microorganisms-3344439-supplementary/Figure S1.png]

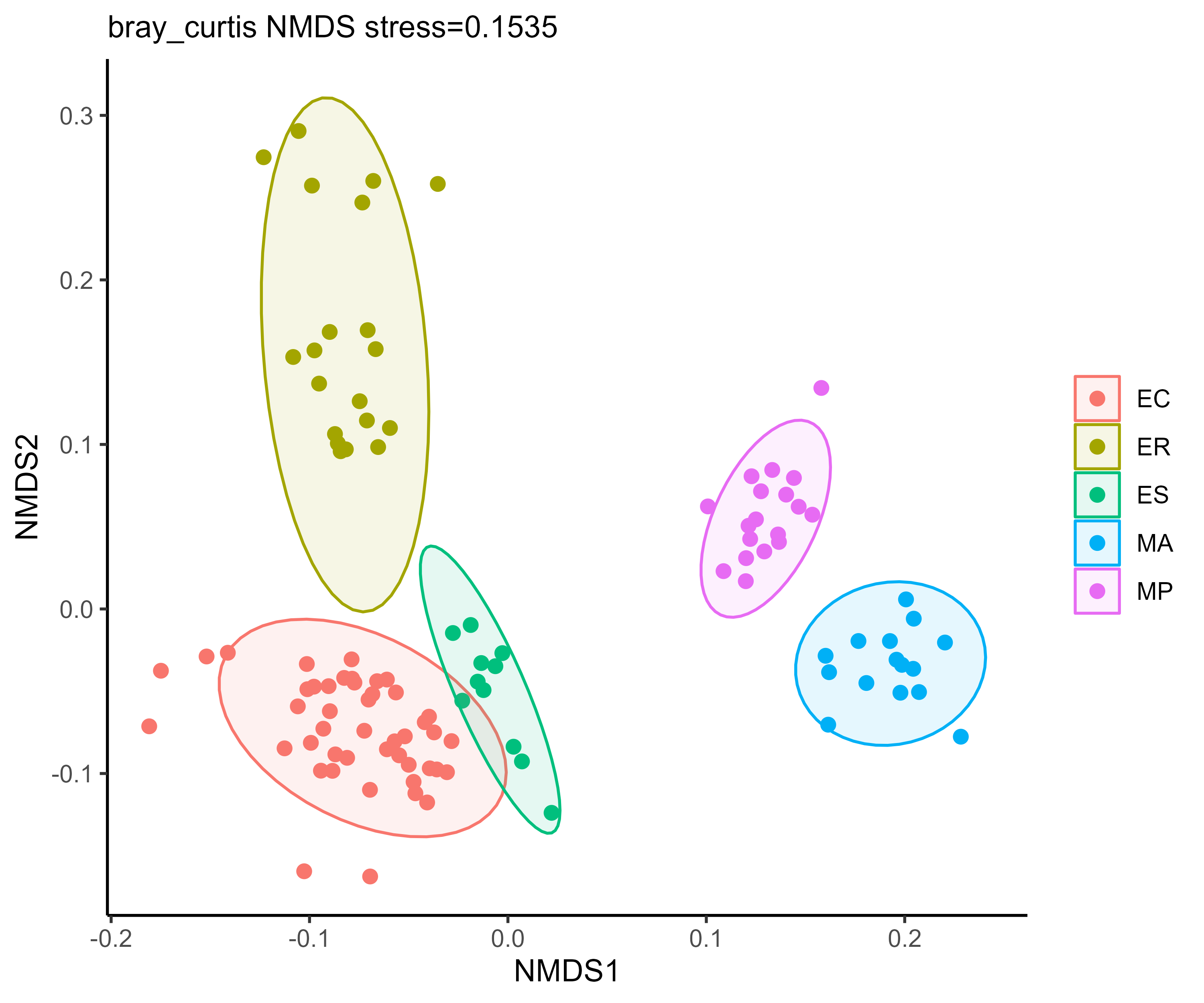

Supplement: Supplementary file 1 [file microorganisms-13-00027-s001.zip › microorganisms-3344439-supplementary/Figure S2.png]

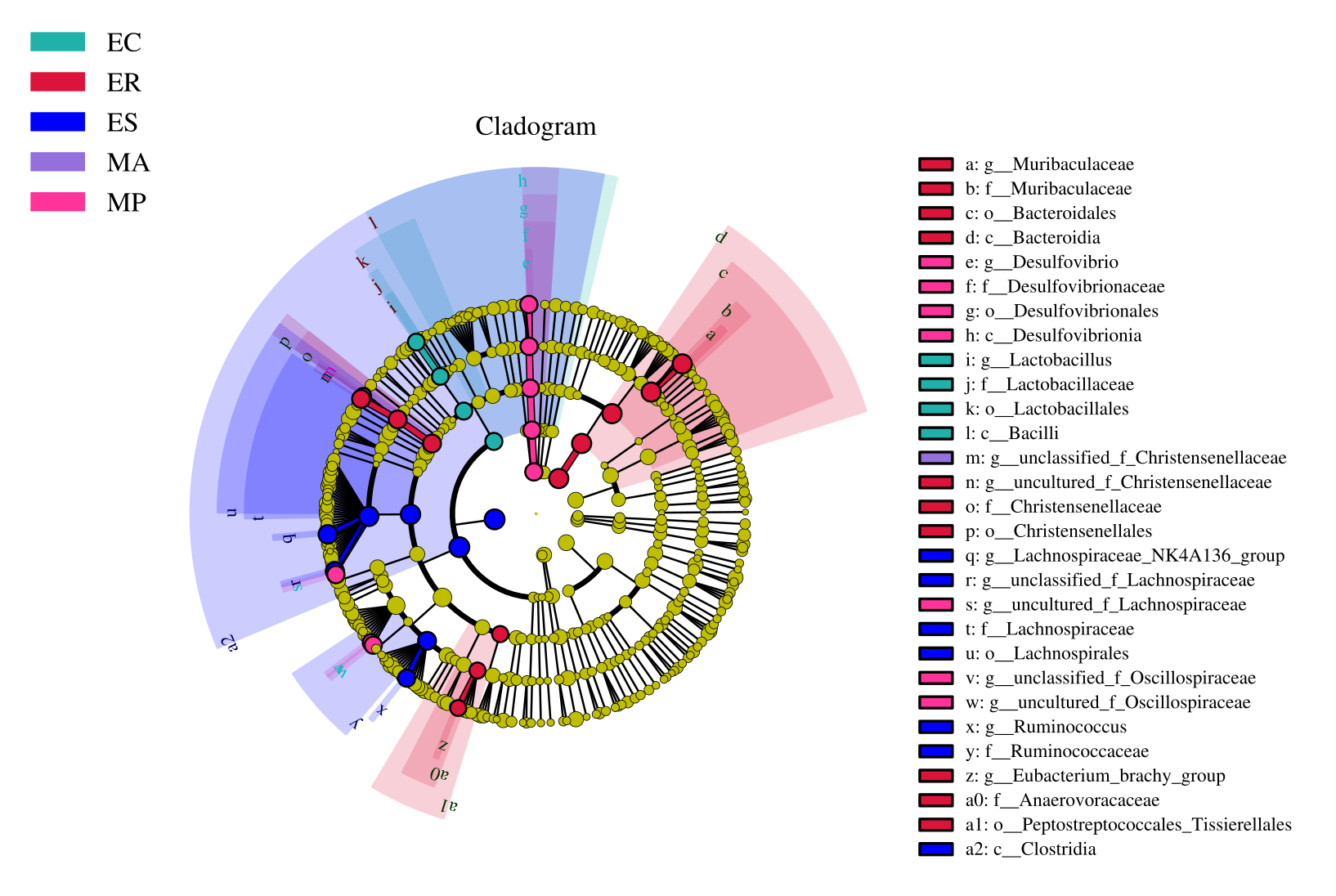

Supplement: Supplementary file 1 [file microorganisms-13-00027-s001.zip › microorganisms-3344439-supplementary/Figure S3.png]

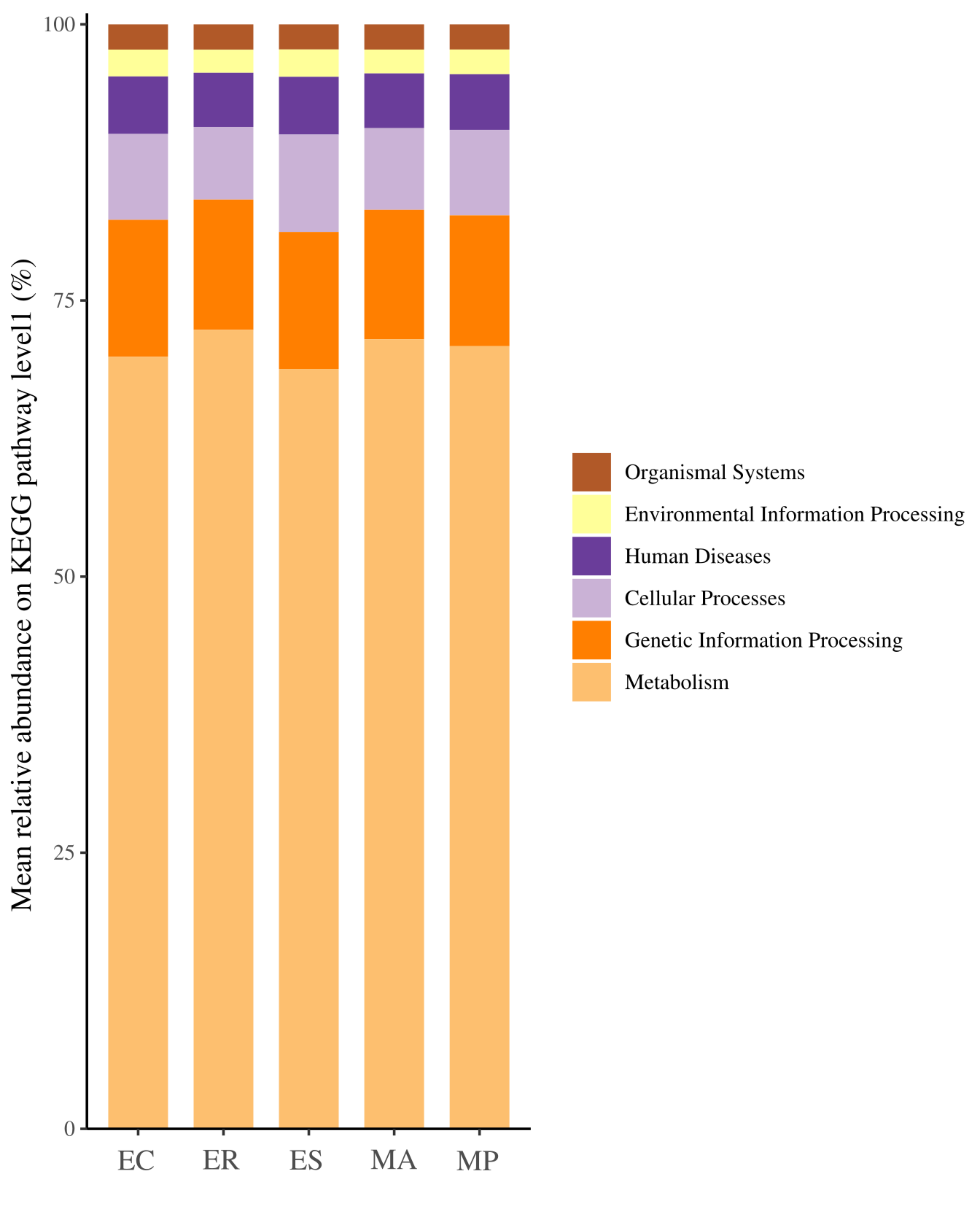

Supplement: Supplementary file 1 [file microorganisms-13-00027-s001.zip › microorganisms-3344439-supplementary/Figure S4.png]

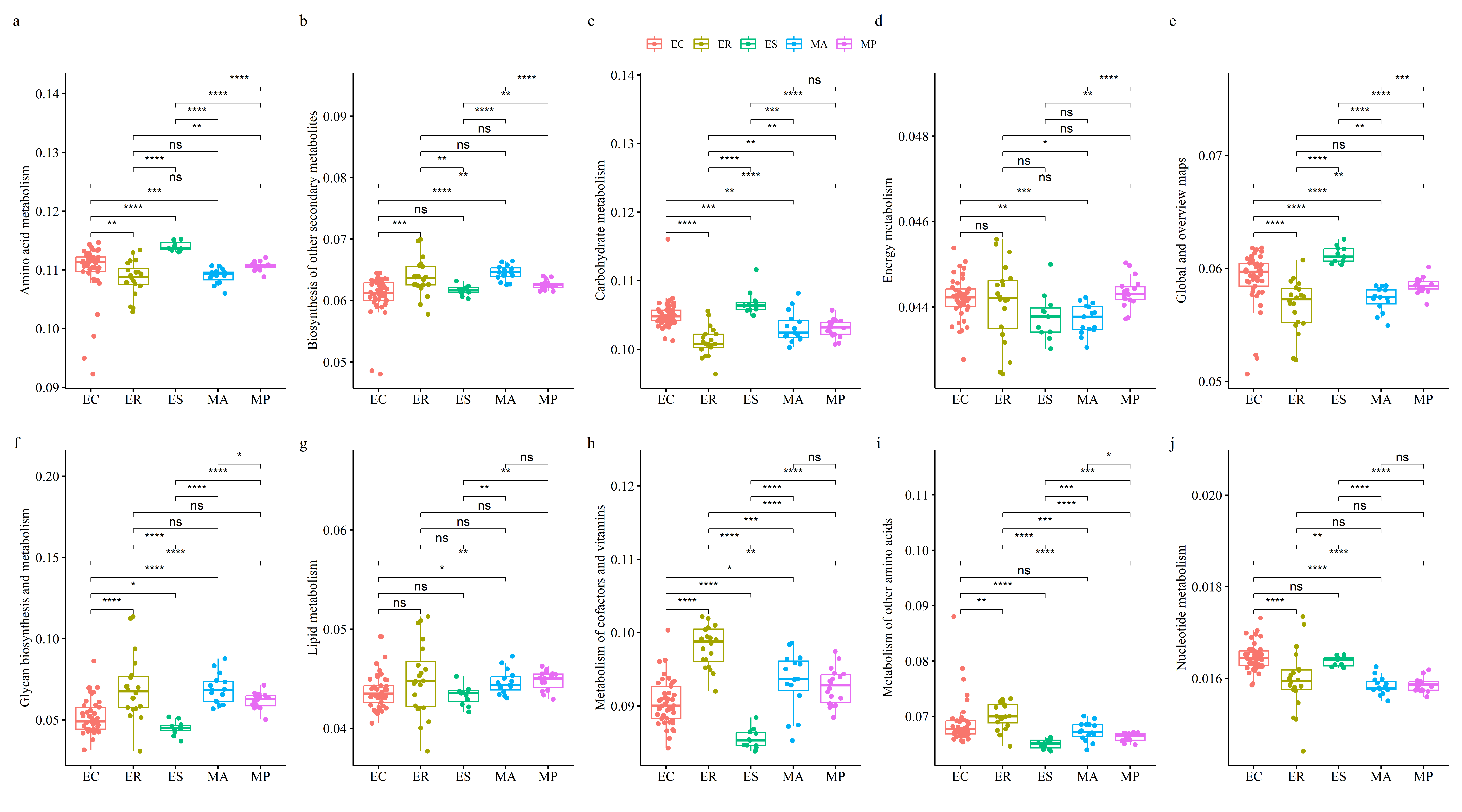

Supplement: Supplementary file 1 [file microorganisms-13-00027-s001.zip › microorganisms-3344439-supplementary/Figure S5.png]
